# Profil épidémiologique des femmes enceintes cardiaques dans le centre de maternité de Tunis: expérience du service A

**DOI:** 10.11604/pamj.2015.21.140.5915

**Published:** 2015-06-22

**Authors:** Khaled Khemiri, Amel Achour Jenayah, Fethia Boudaya, Asma Hamdi, Souad Meskhi, Ezzeddine Sfar, Dalenda Chelli

**Affiliations:** 1 Service de Gynécologie Obstétrique A, Centre de Maternité et de Néonatologie de Tunis, Tunisie

**Keywords:** Cardiopathie, valvulopathies, pronostic, complications, mère, fœtus, Cardiopathy, valvulopathies, prognosis, complications, mothers, fetus

## Abstract

Nous rapportons dans ce travail, le profil épidémiologique des femmes enceintes porteuses d'une cardiopathie ayant accouché dans le service «A» du centre de maternité de Tunis. Il s'agit d'une étude rétrospective descriptive sur 3 ans, de janvier 2010 à décembre 2012, portant sur les dossiers des parturientes cardiaques suivies et ayant accouché dans notre service, à l'exclusion des cardiopathies hypertensives. Les paramètres analysés étaient la gestité et parité des parturientes, le type de la cardiopathie, le mode d'accouchement et le pronostic foetal. Cinquante six cas sur 19655 accouchements avaient été recrutés, soit une incidence de 1 pour 351 accouchements (0,285%). L’âge moyen de nos parturientes était de 30,89±5,3 ans avec des extrêmes allant de 21 à 42 ans. 23 (41,07%) avaient une cardiopathie congénitale, 35 (62,5%) une valvulopathie, 6 (10,71%) un trouble du rythme, 3 (5,35%) un trouble de la conduction à type de bloc auriculo-ventriculaire et 3 (5,35%) une cardiopathie ischémique. Cinquante deux parturientes (92,85%) avaient accouché à un terme dépassant les 37 semaines d'aménorrhée (SA). L'accouchement s'est déroulé par les voies naturelles chez 22 (39,28%) patientes et par césarienne chez 34 (60,71%). Le poids moyen des nouveaux nés à la naissance était de 3341,25 grammes. 3 transferts néonataux en unité de réanimation étaient réalisés avec un seul cas de décès néonatal. La grossesse et l'accouchement chez la femme cardiaque constituent un haut risque materno-foetal dépendant du type, mais surtout du stade de la cardiopathie, nécessitant une prise en charge précoce et multidisciplinaire de la parturiente.

## Introduction

Dans les pays développés la répartition des cardiopathies a connu un changement depuis 30 ans en raison de l'amélioration des conditions sanitaires, de la réduction de l'incidence du rhumatisme articulaire aigu et de l'augmentation de l'espérance de vie avec prédominance des cardiopathies congénitales. Alors que dans les pays en voie de développement y compris la Tunisie, les cardiopathies rhumatismales demeurent dominantes. La grossesse est associée toujours à des adaptations physiologiques et cardiovasculaires importantes. L'association de la grossesse à une cardiopathie maternelle demeure à haut risque et peut être une cause de morbidité voire de mortalité maternelle ainsi que foetale [1]. Notre étude a pour objectif de rapporter le profil épidémiologique des femmes enceintes porteuses d'une cardiopathie, rencontrées dans le service de gynécologie obstétrique A du centre de maternité de Tunis.

## Méthodes

Il s'agit d'une étude rétrospective descriptive sur une période de trois ans allant de janvier 2010 à décembre 2012 menée au service de gynécologie obstétrique A du centre de maternité de Tunis. Toutes les parturientes diagnostiquées porteuses d'une cardiopathie, à l'exclusion des cardiopathies hypertensives ayant séjourné dans le service ont été incluses dans l’étude. Seuls les dossiers incomplets ont été exclus de l’étude. Les données ont été traitées par le logiciel Excel.

## Résultats

**Figure 1:**
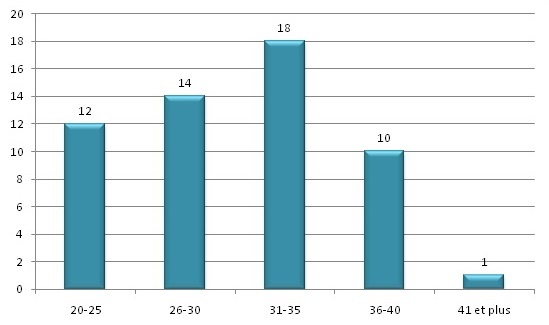
Répartition des patientes selon la tranche d’âge

**Figure 2:**
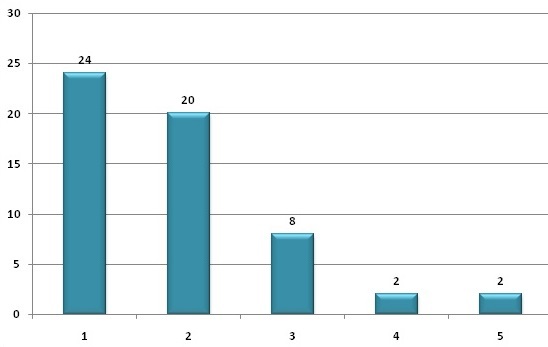
Répartition des patientes selon la parité

Cinquante six cas sur 19655 accouchements avaient été recrutés, soit une incidence de 1 pour 351 accouchements (0,285%). L’âge moyen de nos patientes était de 30,89±5,3 ans avec des extrêmes allant de 21 à 42 ans (Figure 1). La parité moyenne était de 1.91±1,08 avec des extrêmes de 1 et 6. Vingt quatre patientes (42,85%) étaient des primipares (Figure 2). Quatorze patientes (25%) étaient suivies pour un rhumatisme articulaire aigu depuis le jeune âge. La cardiopathie était découverte au cours de la grossesse chez trois patientes. Concernant le type de la cardiopathie, 23 patientes (41,07%) avaient une cardiopathie congénitale, 35 (62,5%) une valvulopathie, 6 (10,71%) un trouble du rythme, 3 (5,35%) un trouble de la conduction à type de bloc auriculo-ventriculaire et 3 (5,35%) une cardiopathie ischémique. Les troubles du rythme cardiaque étaient de type auriculaire dans 4 cas, ventriculaire dans 1 cas et de type Wolf Parkinson White dans 1 cas. Les 3 cas de cardiopathies ischémiques étaient tous des syndromes coronariens. Aucun cas d'infarctus du myocarde n'a été rapporté durant notre période d’étude. Vingt cinq patientes (44,64%) avaient bénéficié d'un geste cardiaque dont 9 cathétérismes, 3 pace maker et 13 cas de chirurgie cardiaque (dont 4 remplacements de la valve mitrale, 1 remplacement de la valve aortique et 1 cas de double remplacement valvulaire mitral et aortique).

Concernant l'accouchement, quarante patientes (71,42%) étaient hospitalisées pour programmation de l'accouchement. Le travail était spontané chez 19 patientes (33,92%) et induit chez 21 (37,1%). Les 16 autres patientes (28,57%) avaient accouché dans un contexte d'urgence. Cinquante deux patientes (92,85%) avaient accouché à un terme dépassant les 37 semaines d'aménorrhée (SA). Nous avons noté 4 (7,14%) accouchements prématurés dont 1 qui est survenu à un terme inférieur à 34 SA. L'accouchement s'est déroulé par les voies naturelles chez 22 (39,28%) patientes et par césarienne chez 34 (60,71%). L'indication de la césarienne était obstétricale dans 15 cas. Deux patientes avaient nécessité un transfert post opératoire au service de réanimation. La première avait un rétrécissement mitral (RM). La seconde avait un rétrécissement aortique (RAo) serré et une insuffisance aortique (IAo) grade 2. Aucun décès maternel n'a été noté dans notre série.

Le poids moyen des nouveaux nés à la naissance était de 3341,25 grammes. Le score d'apgar à la naissance était inférieur à 8 à la première minute chez 3 nouveaux nés. Ces derniers étaient issus d'accouchements par césarienne dont 2 étaient pratiquées dans un contexte d'urgence pour des indications obstétricales. Ces trois nouveaux nés avaient nécessité un transfert au service de néonatologie. Un seul est décédé. Il était né avant 34 SA, et avait pesé 1100 grammes.

## Discussion

Dans cette étude, la fréquence des cardiopathies au cours de la grossesse s'estimait à 0,285 pour cent accouchements. Ce chiffre semble concordant aux données de la littérature. En effet, l'incidence des cardiopathies aiguës au cours de la grossesse est stable entre 1 et 2% depuis les années 1930, avec des estimations plus récentes entre 0,1 et 1,4% aux USA [2]. Notre taux est semblable à Andrianirina [3] (0,2%). Younes rapporte une fréquence un peu plus importante au Maroc (1,4%) [4].

La morbidité maternelle associant insuffisance cardiaque congestive, arythmie et accidents vasculaires est estimée dans la littérature, entre 8 et 30% [2]. Nous n'avons constaté aucune de ces complications chez nos patientes. Concernant le type de cardiopathie: 41,07% de nos patientes avaient une cardiopathie congénitale. Ce taux est franchement au deca de celui avançait par Blackburn [1]: 70 à 80% des cardiopathies rencontrées au cours de la grossesse dans les pays développés sont des cardiopathies congénitales. Cela s'explique par le fait qu'un plus grand nombre de femmes atteintes de CC survivent maintenant jusqu’à l’âge adulte grâce aux techniques chirurgicales et/ou aux traitements pharmacologiques.

Les cardiopathies valvulaires représentaient dans notre série 62,5% des cas (Tableau 1). Elles sont dans l'ensemble bien tolérées, notamment pour les valvulopathies fuyantes, avec une mortalité maternelle nulle. Dans les pays développés, les cardiopathies valvulaires ne concernent pratiquement que des individus migrants, ce qui peut constituer une source de difficultés en raison notamment d'un suivi cardiologique insuffisant, du caractère instable de l'atteinte cardiaque et d'une observance des traitements (particulièrement anticoagulants) aléatoire [2].

**Tableau 1:** Répartition des valvulopathies dans notre série

	Nombre	Pourcentage (%)
**IM**	13	37,14
**RM**	5	8,92
**MM**	2	3,57
**IAo**	1	1,78
**RAo**	2	3,57
**MAo**	3	5,35
**MM+ IAo**	4	7,14
**MAo+ IM**	2	3,57
**IAo+ IM**	2	3,57
**RM+ IAo**	1	1,78

Douze patientes (21,43%) souffraient d'un rétrécissement mitral; isolé dans 5 cas (8,92%) et associé à une insuffisance mitrale dans 2 cas (3,57%) (Tableau 1). Le RM était serré (surface mitrale < 1,5 cm^2^) dans 4 cas. Le rétrécissement mitral est la plus fréquente des valvulopathies. L'augmentation du volume plasmatique (parfois aggravée par des apports liquidiens excessifs en péripartum), la diminution de la pression oncotique plasmatique et la tachycardie qui accompagnent la grossesse créent des conditions favorables à la décompensation, aggravée d'avantage par la perte de la systole auriculaire en cas de passage en fibrillation auriculaire. Un RM serré est associé à une mortalité maternelle de 5%, alors qu'elle est inférieure à 1% dans les formes peu symptomatiques [2]. Une seule patiente ayant un RM (surface mitrale > 1,5 cm^2^) avait été transférée en post opératoire au service de réanimation. Aucun décès maternel chez les patientes valvulopathes n'a été noté dans notre série. Sept patientes (12,5%) souffraient d'un rétrécissement aortique; isolé dans 2 cas (3,57%) et associé à une insuffisance aortique dans 3 cas (5,35%) (Tableau 1). Une patiente ayant une maladie aortique était transférée en post opératoire au service de réanimation.

Quant aux insuffisances mitrales et aortiques, elles sont généralement bien tolérées du fait des modifications physiologiques de la grossesse avec augmentation de la pré-charge et diminution de la post-charge, et n'ont pas de morbi-mortalité associée [2].

Six patientes de notre série, avaient eu un remplacement valvulaire avant la survenue de la grossesse qui était menée sous anti coagulation efficace sans incidents. En effet, le recours à une chirurgie préconceptionnelle peut être nécessaire en cas de risque important de décompensation au cours de la grossesse. La gestion du traitement anticoagulant reste problématique, les antivitamine K exposant au risque d'embryopathie et d'hémorragie du postpartum. Ils restent néanmoins recommandés tout au long de la grossesse devant un risque majoré d’épisode thrombotique sous héparine non fractionnée (HNF) ou héparine de bas poids moléculaire (HBPM). Ce risque étant probablement en rapport avec un sous dosage, certains auteurs proposent un relais par HBPM ou HNF de la 6^e^ à la 12^e^ SA, puis à partir de la 36^ème^ SA autour de l'accouchement [1].

Concernant les troubles du rythme cardiaque, les modifications hémodynamiques et hormonales liées à la grossesse ainsi que l'existence d'une cardiopathie sous-jacente sont les facteurs qui peuvent les favoriser. Les extrasystoles auriculaires et ventriculaires ne nécessitent, généralement, pas de traitement médicamenteux. Néanmoins, il faut identifier et éliminer les éventuels facteurs favorisants [5]. Trois patientes de notre série (5,35%) étaient suivies pour un syndrome coronarien. Elles étaient âgées respectivement de 28, 33 et 39 ans. En fait, l'incidence de la cardiopathie ischémique augmente de part le monde du fait que les femmes ont des enfants à un âge plus tardif et qu'elles sont plus nombreuses à présenter des facteurs de risque de cardiopathie ischémique, incluant l'HTA, l'obésité et le tabagisme [1, 2]. Deux patientes étaient âgées de moins de 35 ans. Ceci rejoint les statistiques allemandes qui rapportant un âge plus jeune de survenue d'ischémie myocardique chez les femmes. La survenue d'IDM chez une patiente est rarissime avec une mortalité maternelle oscillant entre 20 et 37% et foetale de 17% [6].

### Mode d'accouchement et type d'anesthésie

L'accouchement de nos patientes était programmé dans pratiquement les trois quart des cas dont 21 cas (37,1%) de travail induit. Vingt deux patientes (39,28%) avaient accouché par les voies naturelles et 34 (60,71%) par césarienne. Les auteurs sont tous pour l'accouchement par voie vaginale qui demeure le mode d'accouchement de choix pour la plupart des femmes atteintes de cardiopathie, sauf s'il existe des indications obstétriques spécifiques, en présence par exemple d'un risque plus élevé d'infection ou d'une aggravation de la performance cardiaque (modifications hémodynamiques rapides/perte de sang accrue) [1]. Pour ce faire, le mieux habituellement est de laisser le travail commencer naturellement, bien que son induction puisse être appropriée pour optimiser le moment de l'accouchement par rapport à l'administration de l'anticogulation ou aux ressources disponibles [1]. La décision concernant le type d'anesthésie à administrer pour une césarienne sera guidée par la nature et la sévérité de la lésion cardiaque de la mère ainsi que par l'urgence de l'accouchement chirurgical [1].

## Conclusion

La grossesse chez la femme cardiaque est à haut risque materno-foetal. Une planification des naissances avec consultation préconceptionnelle puis un suivi multidisciplinaire impliquant cardiologue, obstétricien, réanimateur, néonatologiste et parfois chirurgien cardiovasculaire sont des mesures nécessaires pouvant garantir une grossesse sans complications.
